# Prophylactic treatment of childhood myopia: a game-changer in combating the scourge of myopia?

**DOI:** 10.3389/fpubh.2025.1556199

**Published:** 2025-07-14

**Authors:** Deborah M. X. Lee, Jonathan T. W. Au Eong, Kah-Guan Au Eong

**Affiliations:** ^1^Department of Cardiothoracic Surgery, Singapore General Hospital, Singapore, Singapore; ^2^Department of General Surgery, Singapore General Hospital, Singapore, Singapore; ^3^International Eye Cataract Retina Center, Mount Elizabeth Medical Center and Farrer Park Medical Center, Singapore, Singapore; ^4^Lee Kong Chian School of Medicine, Nanyang Technological University, Singapore, Singapore; ^5^Department of Ophthalmology and Visual Sciences, Khoo Teck Puat Hospital, Singapore, Singapore

**Keywords:** myopia, premyopia, low-concentration atropine, low-dose atropine, highly aspherical lenslets, defocus incorporated multiple segments

## Introduction

The global prevalence of myopia has been increasing in the last few decades and is projected to reach 50% by the year 2050 in the absence of effective intervention measures (1). Each additional diopter (D) of myopia is associated with increased risk of ocular pathologies such as open-angle glaucoma (20%), posterior subcapsular cataract (21%), retinal detachment (30%), and myopic maculopathy (58%) (2). Hence, there is a pressing need to control myopia progression to reduce the burden of its associated sight-threatening complications.

### Preventive strategies for childhood myopia progression

Until now, the prevention of childhood myopia has been largely focused on secondary prevention, i.e., reducing the severity of the condition for those who already have myopia. Various pharmacologic and optical interventions are currently in use to retard myopia progression (3, 4).

Studies such as the landmark Atropine for the Treatment Of Myopia 2 (ATOM2) (5) and Low-concentration Atropine for Myopia Progression (LAMP) (6) trials in East Asia have provided robust evidence for the effect of atropine on myopia control. The prevailing theory on the mechanism of action is on atropine modulating muscarinic receptors in ocular tissues during development (7). This effect is dose-dependent: the weighted mean differences (WMD) in refraction and axial elongation per year was 0.73 D and −0.26 mm for high-concentration (0.5%−1%) atropine, 0.65 D and −0.37 mm for moderate concentration (0.1%−0.25%), and 0.35 D and −0.11 mm for low-concentration (0.005%−0.05%) (8).

Interestingly, the Atropine for the Treatment Of childhood Myopia in India (I-ATOM) trial on 100 Indian children with mild to moderate myopia found that topical 0.01% atropine reduced refraction progression but had no significant effect on axial elongation (9). The Pediatric Eye Disease Investigator Group (PEDIG) trial on 187 American children with low to moderate myopia found that nightly topical 0.01% atropine did not slow myopia progression or axial elongation after 2 years compared with placebo (10). Change in spherical equivalent refraction (SER) were −0.82 D (atropine) and −0.80 D (placebo; adjusted difference −0.02D; 95% CI, −0.19 to +0.15 D; *p* = 0.83) and axial length (AL) were 0.44 mm (atropine) and 0.45 mm (placebo; adjusted difference −0.002 mm; 95% CI, −0.106 to 0.102 mm). It is worth noting that only a small proportion of participants (11%) in this trial were of East Asian descent, suggesting genetic and cultural or environmental influences on myopia progression.

Balancing effectiveness and potential side effects, topical atropine 0.05% has been deemed to be the optimal concentration for myopia control (8). The commonly reported side effects include photophobia and mild blurry near vision due to pupil dilation and reduced accommodation (11). Most children adapt well over time, and the side effects are reversible upon cessation of treatment (12).

Optical interventions with lenses designed for peripheral myopic defocus have been proven to be effective in signaling the eye to slow down its growth, resulting in slower myopia progression (13). For example, orthokeratology uses myopic shift and peripheral retinal defocus (14) in addition to reshaping the cornea temporarily to achieve good unaided vision during lens-free periods in the daytime (15). Spectacle lenses with highly aspherical lenslets (HAL) (16) and Defocus Incorporated Multiple Segments (DIMS) (17) provide refractive correction for distance vision and peripheral myopic defocus simultaneously by converging some incoming light rays in front of the retina. Cylindrical Annular Refractive Elements (CARE) lenses represent the latest addition to this new generation of myopia control spectacle lenses (18).

### Primary prevention of childhood myopia

Primary prevention of childhood myopia focuses on children who do not yet have myopia (19). Premyopia, as defined by the International Myopia Institute, is a refractive state between +0.75 and −0.50 D in children with risk factors such as increased AL, myopic parents, or environmental influences (20). While no strict age cutoff is given, children are considered at higher risk if their refractive error is < +0.75 D at age 6, ≤ +0.50 D at ages 7–8, and ≤ +0.25 D at ages 9–10 (21). Current practices rely solely on behavioral modification to delay the onset of myopia in premyopes but we posit that in high risk groups, supplementing this with prophylactic treatment using pharmacologic and/or optical interventions may increase the efficacy of myopia control.

#### Behavioral modification

Outdoor time and sunlight exposure have been hypothesized to prevent myopia development by stimulating light-sensitive dopamine release in the retina (22). Dopamine inhibits ocular axial elongation, the anatomical basis of myopia development (22). This relationship has been proven in several studies (23–25), including a 3-year cluster-randomized trial in Guangzhou, China, which demonstrated a 9.1% absolute reduction in myopia incidence and 23% relative reduction over 3 years with 40 min of outdoor activity added per school day (39.5% incidence in control group vs. 30.4% in intervention group, *p* < 0.001) (24). Reduction of near work and longer viewing distances also exert a protective effect against myopia onset by reducing variation in accommodative demand and promote more uniform levels of retinal focus (25–27).

#### Prophylactic treatment of childhood myopia

Treating myopia before its onset in premyopes can potentially further reduce the risk of myopia-associated morbidities than current preventive strategies. Among the modalities, low-concentration atropine is currently the most evidence-supported intervention.

##### Pharmacologic intervention

Using topical low-concentration atropine in premyopes is a novel primary prevention strategy (28). A recent meta-analysis of three randomized controlled trials (RCT) and one non-RCT involving a total of 644 premyopic children aged 4–12 years highlighted its effectiveness in reducing rapid myopic shift (≥0.5 D/year, *p* < 0.04) and myopia incidence (*p* = 0.03) with 12–24 months of use as compared to placebo (Table 1) (28).

**Table 1:** Summary of results from a meta-analysis of three randomized controlled trials (RCT) and one non-RCT on the effectiveness of low-concentration atropine vs. placebo in reducing myopia incidence, rapid myopia shift, refraction (spherical equivalent, D) and axial elongation (mm) in premyopic children (28).

**Outcome**	**Duration**	
	**6–12 Months**	**12–24 Months**
Myopia incidence (RR, 95% CI)	0.48 (0.22–1.01), *p* = 0.05	0.62 (0.40–0.97), *p* = 0.03
Rapid myopic shift ≥0.5 D/year (RR, 95% CI)	0.58 (0.39–0.86), *p* < 0.01	0.50 (0.26–0.96), *p* < 0.04
Mean difference in spherical equivalent (D) (WMD, 95% CI)	0.31 (0.16–0.47), *p* < 0.01	0.58 (0.18–0.98), *p* < 0.01
Mean difference in axial elongation (mm) (WMD, 95% CI)	−0.10 (−0.15 to −0.06), *p* < 0.01	−0.19 (−3.00 to −0.07), *p* < 0.01

##### Optical intervention

Evidence is now emerging that lenses with HAL and DIMS may be useful in treating premyopes. A RCT with 108 low-hyperopic (0 to +2.00 D) Chinese children aged 6–9.9 years demonstrated significantly slower axial elongation (*p* < 0.001) with HAL lenses (0.11 mm; interquartile range [IQR]: 0.05–0.17 mm) compared to single vision lenses (0.27 mm; 0.21–0.33 mm) (29). A dose-response relationship was observed, with >30 h of wear per week proving efficacious (29). DIMS lenses were also shown to stabilize progression in SER (+0.04 D) and axial elongation (+0.06 mm) over 3 months of usage of ≥10 h/day in a pilot study involving 24 premyopic Taiwanese children aged 5–6 (30). Comparatively, mean axial elongation in a 6-year-old Chinese population was 0.58 mm/year (99% confidence interval [CI] 0.56–0.59 mm/year) in myopes and at 0.65 mm/year (99% CI 0.62–0.67 mm/year) in incident myopes (31). Over 9 months of DIMS lenses use, the average cycloplegic SER remained stable with a yearly change of +0.06 D compared to −0.15 D in a control group (32). There are currently no published studies specifically evaluating the use of orthokeratology lenses in premyopic children.

### A game changer?

We are excited by the findings of these recent studies and believe that the prophylactic treatment of childhood myopia could be a game-changer in combating the scourge of myopia. Myopia control interventions employed in myopic children typically reduces axial elongation by < 0.4mm compared with control groups within a 2-year study period (29). In contrast, delaying myopia onset from age 9–10 is estimated to reduce axial elongation into adulthood by around 0.5 mm, suggesting that preventing or delaying onset may have a greater long-term impact (29).

### Challenges in starting prophylactic treatment of myopia

Currently, topical low-concentration atropine therapy and optical interventions for prophylactic myopia treatment are not yet part of routine clinical practice (33, 34). One possible concern is the main side effect of atropine use—photophobia, although low atropine concentrations do not significantly affect vision-related quality of life (*p* > 0.05) (35). Simple measures such as wearing sunglasses outdoors, using photochromic lenses (36), or administering atropine eye drops at bedtime can ameliorate this symptom.

Wearing myopia control spectacles can lead to concerns about appearance, discomfort, and inconvenience—such as slipping, fogging, or pressure on the nose bridge during daily activities and sports (37). Seventeen per cent of premyopic children reported inconvenience to their daily activities at home and 8.3% had eye strain using DIMS lenses, although the frequency and severity of the complaints were mild (30). These factors can make optical interventions challenging to adopt in premyopic children who have good unaided vision.

There is currently a gap in public awareness about pharmacologic and optical intervention for primary myopia prevention, with few seeking treatment at a premyopic stage as vision is not problematic yet (38). Additionally, starting prophylactic myopia treatment may lead to greater financial burden as treatment, follow-up and opportunity costs start accumulating at a younger age (39, 40).

Despite these caveats, prophylactic treatment appears to be a promising novel strategy to delay the onset of myopia. Combined with secondary prevention, they aim to ultimately reduce the prevalence of high myopia and its associated sight-threatening complications.

### Further research

Further studies on different topical atropine concentrations for primary myopia prevention can guide clinicians on the optimal concentration in this population. Investigating synergistic treatment such as low-concentration atropine and myopia control spectacle lenses can aid in proposing solutions for children intolerant to higher doses. Lastly, by developing multifactorial risk scores for premyopia for patient selection and better public education, we can hopefully see a large benefit in this new approach to preventing myopia-related complications and improving long-term outcomes.

## Conclusions

Primary prevention of myopia with topical low-concentration atropine has recently been proven effective in delaying myopia incidence by reducing rapid myopic shift in SER and axial elongation. While we await future research to shed more light on prophylactic interventions, current evidence suggests that incorporating prophylactic treatment of myopia in premyopic children into current myopia control strategies could potentially be a game changer in combating the scourge of myopia.
